# Chickpea Genotypes Contrasting for Vigor and Canopy Conductance Also Differ in Their Dependence on Different Water Transport Pathways

**DOI:** 10.3389/fpls.2017.01663

**Published:** 2017-09-26

**Authors:** Kaliamoorthy Sivasakthi, Murugesan Tharanya, Jana Kholová, Ruth Wangari Muriuki, Thiyagarajan Thirunalasundari, Vincent Vadez

**Affiliations:** ^1^Crop Physiology Laboratory, International Crops Research Institute for the Semi-Arid Tropics, Pantancheru, India; ^2^Department of Industrial Biotechnology, Bharathidasan University, Tiruchirappalli, India; ^3^Department of Agriculture, Egerton University, Egerton, Kenya

**Keywords:** hydraulic conductance, transpiration rate (TR), aquaporins, apoplastic pathway, water deficits, high vapor pressure deficit (VPD), vigor

## Abstract

Lower plant transpiration rate (TR) under high vapor pressure deficit (VPD) conditions and early plant vigor are proposed as major traits influencing the rate of crop water use and possibly the fitness of chickpea lines to specific terminal drought conditions—this being the major constraint limiting chickpea productivity. The physiological mechanisms underlying difference in TR under high VPD and vigor are still unresolved, and so is the link between vigor and TR. Lower TR is hypothesized to relate to hydraulic conductance differences. Experiments were conducted in both soil (Vertisol) and hydroponic culture. The assessment of the TR response to increasing VPD showed that high vigor genotypes had TR restriction under high VPD, and this was confirmed in the early vigor parent and progeny genotype (ICC 4958 and RIL 211) having lower TR than the late vigor parent and progeny genotype (ICC 1882 and RIL 022). Inhibition of water transport pathways [apoplast and symplast (aquaporins)] in intact plants led to a lower transpiration inhibition in the early vigor/low TR genotypes than in the late vigor/high TR genotypes. De-rooted shoot treatment with an aquaporin inhibitor led to a lower transpiration inhibition in the early vigor/low TR genotypes than in the late vigor/high TR genotypes. Early vigor genotypes had lower root hydraulic conductivity than late vigor/high TR genotypes. Under inhibited conditions (apoplast, symplast), root hydraulic conductivity was reduced more in the late vigor/high TR genotypes than in the early vigor/low TR genotypes. We interpret that early vigor/low TR genotypes have a lower involvement of aquaporins in water transport pathways and may also have a smaller apoplastic pathway than high TR genotypes, which could explain the transpiration restriction under high VPD and would be helpful to conserve soil water under high evaporative demand. These findings open an opportunity for breeding to tailor genotypes with different “dosage” of these traits toward adaptation to varying drought-prone environments.

## Introduction

Chickpea (*Cicer arietinum* L.) is the second most important legume crop after the dry bean worldwide (FAOSTAT, 2014). It is grown on low input marginal lands and represents an important component of subsistence farming. Chickpea is mostly grown on residual soil moisture from monsoon rain on the Indian sub-continent and semi-arid regions of sub-Saharan Africa (SSA). Therefore, terminal (end season) drought stress in chickpea is the major constraint for yield loss (Krishnamurthy et al., 2010), which causes typical yield losses upto 50% (Ahmad et al., 2005).

Plant adaptations to cope with end-season water deficit revolve around the need to use water in an efficient way—i.e., to ensure that water is available for grain filling period (Vadez et al., 2013), and water management is tightly dependent on canopy vigor and transpiration rates. Our recent work documented that lower canopy conductance [TR (mg H_2_O cm^−2^min^−1^)] under high vapor pressure deficit (VPD) conditions but with no soil water limitation could contribute to the terminal drought adaptation in chickpea (*Cicer arietinum* L.; Zaman-Allah et al., 2011a). In crops grown on residual soil moisture, this is one of the mechanisms that allows to conserve water in the soil profile during early plant development and use the retained water later in the season for grain filling. This mechanism has been described in other species like pearl millet (*Pennisetum glaucum* L.; Kholova et al., 2010), sorghum (*Sorghum bicolor* L.; Gholipoor et al., 2010; Kholova et al., 2014), soybean (*Glycine max* L.; Merr; Fletcher et al., 2007; Gilbert et al., 2011), peanut (*Arachis hypogaea* L.; Devi et al., 2010), cowpea (*Vigna unguiculata* L.; Belko et al., 2012), and in maize (*Zea mays* L.; Yang et al., 2012; Gholipoor et al., 2013). In addition, Zaman-Allah et al. (2011a) also hypothesized that retaining water at vegetative stage could be the consequence of low early vigor (slower early development of leaf area and above-ground biomass) and showed that terminal drought-tolerant lines had indeed low early vigor.

An earlier study shows the possibility of a link between high early vigor (rapid early development of leaf area and above-ground biomass) and transpiration sensitivity to increasing VPD. The above described differences in canopy transpiration response to increasing VPD may also relate to a tight regulation of the plant hydraulic conductivity. Radial water uptake in the root cylinder has been shown to restrict the overall plant hydraulics (Chaumont et al., 2005; Sivasakthi et al., in communication). Radial root hydraulic conductivity (*Lp*) of roots is highly variable among species, genotypes, and root types (Vandeleur et al., 2014; Sivasakthi et al., in communication). Radial water transport through the root cylinder is described by the composite transport model (Steudle and Peterson, 1998), proposing that water flows across the root cylinder via two main pathways, i.e., the apoplastic and symplastic (or cell-to-cell) pathways, toward the xylem: . The apoplastic pathway is considered as a water movement through capillary spaces in the cell walls, whereas in the cell-to-cell pathway, water moves across membranes mainly by facilitated diffusion through water channels, i.e., through aquaporins (AQP and also through plasmodesmata (Knipfer and Fricke, 2010). AQP are water-channeling proteins that facilitate the transport of water molecules across biological membranes (Johansson et al., 2000; Li et al., 2014). The radial root water flow is driven by pressure gradients (hydrostatic and osmotic), encountering a major hydraulic resistance at the endodermis where the apoplastic water movement is limited by casparian bands and water molecules may have to enter the cell-to-cell pathway. Water movement by the cell-to-cell pathway is considered to be mainly driven by osmotic gradients (Steudle, 2000). The biological significance of aquaporins in plants is their ability to modulate transmembrane water transport in situations where adjustment of water flow is physiologically critical (Baiges et al., 2002; Luu and Maurel, 2005). Aquaporin activity may then finely regulate the rate of water flow across the root through gating and modification of their abundance (Bramley et al., 2009).

There are documented interspecific differences in the proportion of water transported by each pathway; e.g., the aquaporin-mediated pathway is hypothesized to predominate in crops like barley (Knipfer et al., 2011), whereas the apoplastic pathways is hypothesized to predominate in crops like maize (Zimmermann and Steudle, 1998). In addition, the intraspecific differences in particular pathways conductivity have been shown in relation to differential water usage strategies (e.g., maize, sorghum—(Choudhary et al., 2013, 2015); soybean—(Sadok and Sinclair, 2010); peanut—Devi et al., 2012). Therefore, here, we tested the hypothesis whether the differences in water transport pathways through the root (aquaporins/apoplast) could explain the differences in the transpiration responses to increased VPD. The approach is then to follow the transpiration response to the inhibition/blockage of the water transport pathways. Water flow through the aquaporins is inhibited with AQP-specific molecules, such as HgCl_2_, AgNO_3_, and H_2_O_2_. Water flow through the apoplast is altered using perfusion techniques in root where insoluble minerals are allowed to sediment in the apoplast pathway, leading to its partial blockage (Ranathunge et al., 2004). This work was carried out in genotypes contrasting for the transpiration response to increasing VPD, i.e., contrasting parents and recombinant inbred lines (RIL).

The objectives of the study were to (i) assess the putative relationship between early plant vigor differences and the capacity to restrict transpiration under high VPD, (ii) test if chickpea genotypes contrasting in their transpiration rate (TR) response to increasing VPD differ in the degree of inhibition of water transport pathways (aquaporin-mediated and apoplastic) in either whole plants or de-rooted shoot, and (iii) assess root hydraulic conductivity in these genotypes prior and after inhibition of water transport pathways.

## Materials and methods

### Plant materials and growth conditions

A pair of parental chickpea genotypes ICC4958 (with an early development of a large root and shoot system, i.e., early vigor) and ICC1882 (with an early development of a small root and shoot system, i.e., late vigor) contrasting for plant vigor were selected (Kashiwagi et al., 2005, 2006). These two genotypes were obtained from ICRISAT mini-core collection and both are desi types. More recently, these two genotypes were found to be contrasting in their transpiration response to natural changes in atmospheric VPD (Sivasakthi et al., 2017). These two genotypes were also found to be contrasting for root traits, and these two contrasting materials (ICC4958 × ICC1882) had also been used to develop 232 recombinant inbred lines (RILs) (Kashiwagi et al., 2005). The entire population of RILs [232 (progenies) +2 (parents)] was screened for transpiration response to natural changing atmospheric VPD under outdoor conditions (Muriuki et al., unpublished data). In short, plants were grown in 8” plastic pots filled with 5 kg black soil (four replications per genotype). Plants were grown under well-watered conditions outdoors. The day before the experiment, the pots were irrigated and left to drain overnight to reach field capacity. A plastic sheet was put on top of the soil and then covered by a 2 cm layer of plastic beads to limit soil evaporation. This system was shown to limit 95% of soil evaporation (more details in Ratnakumar and Vadez, 2011; Vadez et al., 2013; Kholová et al., 2016). All the pots were weighed three times at 7:00, 10:30, and 14:30 h during the day to measure plant transpiration (see details of screening method in Kholová et al., 2012; Kakkera et al., 2015). On the basis of this screening, the 20 most contrasting materials for TR were selected [10 lines with lowest TR values (211, 207, 221, 199, 52, 46, 34, 128, 125, and151) and 10 lines with highest TR values (224, 20, 35, 161, 217, 170, 90, 144, 123, and 22)]. In addition, the whole population was screened in the LeasyScan phenotyping platform (Vadez et al., 2015) for plant vigor-related traits (Sivasakthi et al., unpublished data). From this experiment, plant vigor score data of the above selected lines were retrieved and presented here. The selected 20 entries were grown in glasshouse and the transpiration was measured under increasing VPD conditions in growth chamber (Conviron-PGW36 model, Controlled Environments Limited, Winnipeg Manitoba, Canada: see more details in http://www.conviron.com/sites/default/files/PGW36%20Data%20Sheet_1.pdf). Based on these experimental results, the two most contrasting RILs for TR were selected (one low TR line having early vigor and another one high TR line having late vigor) along with the two parental genotypes. Therefore, two genotypes with low TR and early vigor (ICC 4958 and RIL 211) and two genotypes with high TR and late vigor (ICC 1882 and RIL 022) were selected for further assessment and comparison of water transport pathways and hydraulic properties.

Five groups of experiments were conducted to test (1) the assessment of TR under high VPD conditions in 20 contrasting RILs, (2) the response of the selected two most contrasting RILs and parents for the TR response to increasing VPD, (3) the effect of the inhibition of water transport pathways on the transpiration in whole plants, (4) the measurement of root hydraulic conductivity with or without inhibition or blockage of the water transport pathways, and (5) the effect of the inhibition of water transport pathways on the transpiration of de-rooted shoot. Experiments 3, 4, and 5 were carried out with the two most contrasting RILs and parents. In Experiments 1, 2, and 5, plants were grown in black soil (Vertisol) and remaining experiments (Experiments 3 and 4) were grown in hydroponics system (details in Supplementary Table 1). For all experiments, plants were grown in glasshouse and shifted to the growth chamber a day before the actual experiment to allow plant acclimation. The day and night temperatures and relative humidity (RH %) were on average 28/22°C and 70/90%, respectively, and were under natural day-light oscillations.

In Experiments 1, 2, and 5, plants were grown in 8” plastic pots filled with 5 kg black soil (Vertisol) collected from the ICRISAT farm and fertilized with DAP (di-ammonium phosphate) at the rate of 0.3 g per kg of soil. The top soil of each pot was added with 0.3 g of carbofuran one day before sowing to prevent soil-borne pests. Seeds were treated with fungicides (Thiram®; Sudhama Chemicals Pvt. Ltd. Gujarat, India) to avoid fungal contamination. Four seeds were sown in each pot, and a rhizobium inoculum (Strain No: IC 2002) was added to each pots to ensure proper nodulation. Two weeks after sowing, plants were thinned to two plants per pot. Plants were grown under well-watered conditions up to 32 days. The data logger (Lascar Electronics Inc. UK) was positioned within the plant canopy in the growth chamber for regular records of the air temperature and relative humidity throughout the measurement period.

### Response of transpiration rate (TR) to increasing VPD

For Experiments 1 and 2, assessment of the transpiration response to increasing VPD was performed at 32 day after sowing (DAS) when plants were at vegetative stage in a growth chamber (controlled conditions). One day before the experiment, pots were irrigated and allowed to drain overnight. A plastic sheet was placed on top of the soil and then a 2 cm layer of plastic beads were put on top of the sheet to limit soil evaporation. Plants were moved to the growth chamber where their transpiration response to increasing VPD was assessed under controlled conditions. The plants got acclimatized the day before experiment to day/night VPD conditions of 1.8 kPa (31°C and 60% RH) and 0.9 kPa (27°C and 75% RH), respectively. A light intensity of 450 μmol m^−2^ s^−1^ was measured at the canopy level. During the acclimation day, a day VPD of 1.8 kPa was maintained from 06:30 to 18:30 and the night VPD of 0.9 kPa was maintained from 19:30 to 05:30. The 1-h time gap between day and night regime was used for a progressive transition between VPDs. On the day of the experiment, VPD was gradually increased (0.9–4.21kPa, Supplementary Table 2) in 60-min interval during eight consecutive hours (8:00–15:00 h.). Plants were weighed gravimetrically once in every 60 min, at the beginning of each VPD step, with 0.01 g precision scales (KERN 3600-2N, Kern & Sohn GmbH, Balingen, Germany) to derive transpiration values from consecutive weighings. Between successive VPD levels, 15 min transition was allowed to gradually increase the VPD to the next level. A data logger (Lascar Electronics Inc. UK) was positioned within the plant canopies in the growth chamber for regular records of the air temperature and relative humidity throughout the measurement period. Plants were harvested at the end of the transpiration measurement. Detached leaflets were arranged in the transparent plastic sheets and leaf area was measured by leaf area meter (LI-3100C area meter, LI-COR®Biosciences, and USA).

Experiments 3 and 4 were carried out to test the effect of different inhibitors of the symplastic or apoplastic pathway on plant transpiration and to investigate plant hydraulic characteristics. For this, the plants were grown in a hydroponics system. Seeds were treated with Thiram®; (Sudhama Chemicals Pvt. Ltd. Gujarat, India) to avoid fungal contamination and ensure good germination. Seeds were sown in sand saturated with nutrient solution and rhizobium inoculum (Strain No: IC 2002) was added to ensure proper nodulation. A week after sowing, the seedlings were transferred to 250 ml Erlenmeyer conical flask containing nutrient solution (modified Hoagland solution). The composition of the solution was MgSO_4_ (1 mM), K_2_SO_4_ (0.92 mM), CaCl_2_.2H_2_O (0.75 mM), KH_2_PO_4_ (0.25 mM), Fe-EDTA (0.04 mM), Urea (5 mM), and micronutrients [H_3_BO_3_ (2.4 μM), MnSO_4_ (0.9 μM), ZnSO_4_ (0.6 μM), CuSO_4_ (0.62 μM), and Na_2_MoO_4_ (0.6 μM)]. The pH of the nutrient solution was adjusted between 6.0 and 6.2. Seedlings were passed carefully through the hole of a rubber stopper that fitted tightly to the conical glass flask aperture and the hypocotyle was fixed with cotton to avoid the seedling from slipping through. The flasks were painted with two layers: first layer with black paint to ensure darkness in the rooting medium and to prevent the algae growth followed by a second layer of white paint to reflect the excess sun rays and to avoid over-heating of roots. Aeration was continuously supplied to roots by using compression pump (Oil free Air Compressor-CPM 7.5 D TM, Chicogo Pnematic http://www.cp.com, India). Refilling of the flasks were done daily with deionsed water to compensate water losses and the nutrient solution in the flasks was changed every 3 days once.

### Whole plant inhibitions by AQP inhibitor

For this experiment, plants were grown in hydroponics and shifted to the growth chamber for acclimation (experimental conditions: 31°C/60% RH, 450 μmol m^−2^ s^−1^ photosynthetic photon flux density during the day and 25°C/77% RH, 0 μmol m^−2^ s^−1^ photosynthetic photon flux density during the night) one day before the inhibition experiment. The following morning, a measured amount of fresh nutrient solution was given to the plants. Care was then taken to avoid direct evaporation of the hydroponics solution by sealing the corks holding the plant into the flask with aluminum foil. Each single plant was positioned on separate 0.01 g precision scales (KERN 3600-2N, Kern & Sohn GmbH, Balingen, Germany) and their weight loss (due to transpiration) was assessed every 30 min. The aquaporin inhibitor (20 μM HgCl_2_, 50 μM AgNO_3_, and 1 mM-H_2_O_2_) was applied 2 h after the beginning of weight recording for transpiration assessment and it was continued for a minimum of 3 h after treatment. At the end of the inhibition treatment with 50 μM-AgNO_3_, canopy temperature was also measured in addition to transpiration. Canopy temperature was used here as an approximate proxy for the degree of transpiration inhibition in the tested genotypes. The canopy temperature was recorded on 6 replicated plants for each of the control and treatment (inhibited) plants in growth chamber at 3.1 kPa. Thermal images were taken with an infrared (IR) FlexCam S (Infrared Solutions, Plymouth, MN, USA) with a sensitivity of 0.09°C and an accuracy of ±2%. SmartView 2.1.0.10 software (Fluke Thermography Everett, WA, USA) was used for the analysis of the thermal images and the estimation of canopy temperatures.

The aquaporin inhibitor concentration were standardized in earlier experiments (data not shown). Three types of AQPs inhibitors [HgCl_2_, AgNO_3_and H_2_O_2_] were used with various concentrations in this study. The standardization of aquaporin inhibitor concentration for HgCl_2_, AgNO_3_, and H_2_O_2_was done several times (in case of HgCl_2_ 200 μM to 10 μM; AgNO_3_ 400 μM to 25 μM; H_2_O_2_ 2 mM to 1 mM). From these preliminary experiments, 20 μM HgCl_2_, 50 μM AgNO_3_, and 1 mM-H_2_O_2_ were chosen as adequate concentration as these concentrations showed clear genotypic difference in tested genotypes and did not appear to be deleterious to the plants (data not shown).

### De-rooted shoot inhibition by AQPs inhibitor

For this experiment (Experiment 5), soil grown plants were used and assayed at 32 days after sowing. Plants were acclimated to the growth chamber conditions as in Experiment 1. The following morning, plants were cut at the hypocotyl level and the cut end of the de-rooted shoot was immersed in deionized water. Then a single re-cutting was done under 0.1 mM di-sodium EDTA to prevent xylem vessel damage. Brown colored glass containers containing 250 ml of 0.1 mM EDTA were prepared earlier and the mouths of the containers were wrapped with laboratory film (Para film “M”®, Bemis Flexible Packaging, Neenah, WI). The de-rooted shoots were inserted into the flasks by piercing the para film. Finally, the mouths of the flask were wrapped with aluminum foil to limit direct evaporation of water. A small hole was made in the aluminum foil to avoid negative pressure created in the flasks due to removal of water by transpiration. The de-rooted shoots were acclimatized in the growth chamber for 3 h, which was the shortest recovery period necessary for the shoots to attain a stable transpiration (data not shown). Initially, the shoots were acclimatized at low VPD (25°C and 80% Rh, 0 μmol m^−2^ s^−1^ photosynthetic photon flux density) and dark conditions for an hour. Later on, the VPD and light intensity were gradually increased upto 2.5 kPa, i.e., 35°C and 55% RH, 450 μmol m^−2^ s^−1^ photosynthetic photon flux density, and maintained at 2.5 kPa VPD throught out the day. Transpiration was measured gravimetrically with 0.01 g precision scales every 30 min manually. After the initial assessment of transpiration for 2 h, the AQP inhbitors (50 μM HgCl_2_) were applied to the derooted shoots, and the assessment on transpiration continued for 4 h following the treatment. For the untreated control, five replications/genotype were used, and for the treatment, six replications/genotype were used (Supplementary Table 1). Shoots of all plants were harvested at the end of the transpiration measurements. Detached leaflets were arranged in the transparent plastic sheets and leaf area was measured by leaf area meter (LI-3100C area meter, LI-COR®Biosciences, and USA).

### Whole plant inhibition by apoplastic inhibitor

For this experiment (Experiment 3b), 25 days old hydroponically grown plants were used to test the effect of apoplastic pathway inhibition on the plant transpiration. Apoplastic inhibitions consists of precipitates of insoluble inorganic salts that are used to block the extra-cellular pathway. The reaction between 1 mM K_4_[Fe(CN)_6_] and 0.25 mM CuSO_4_ gives rusty brown crystals (precipitates) of Cu_2_[Fe(CN)_6_]_6_ or Cu[CuFe(CN)_6_] (Ranathunge et al., 2005). As CuSO_4_ permeates faster than K_4_ [Fe (CN)_6_], standardization was done to optimize the time interval between successive additions of both the chemicals. A 3 h exposure of the plants to 1 mM K_4_ [Fe (CN)_6_] and solution exchange with 0.25 mM CuSO_4_ was found to give clear genotypic difference for all the tested genotypes.

### Root hydraulic conductivity assessments

In Experiment 4, hydroponically grown plants were used to measure root hydraulic conductivity with help of a pressure chamber (PMS instruments, Corvallis, Oregon, USA) using protocols similar to those described in barley (Tazawa et al., 1997), wheat (Maggio and Joly, 1995), tomato (Zhang and Tyerman, 1999), and pearl millet (Tharanya et al., 2017). This measurement was performed under glass house conditions, and this was done in plants that were previously treated with either aquaporin (20 μM- HgCl_2_) or apoplast inhibition (1 mM K_4_[Fe(CN)_6_] and 0.25 mM CuSO_4_) or under non-treated conditions. The shoot was cut using a razor blade. The detached root, which was bathing in solution (deionized water in case of untreated control and deionized water plus inhibitors in case of treatment), was carefully placed in the pressure chamber and sealed using silicon glue and polyvinylsiloxane (Coltene President Company, Switzerland) to prevent pressure leakage. The pressure levels (0.1, 0.2, and 0.3 MPa) were successively applied in the root medium, each being maintained for 15 min. The root exudate (xylem sap) for each pressure was collected thrice (every 5 min once) at the cut surface using pre-weighed eppendorf stuffed with tissue paper (Kimtech Science, Ontario, USA). After a constant exudation rate was reached at each pressure, the next level of pressure was applied. The average value of the three exudation samples was normalized with root surface area, pressure, and time. The root surface area was estimated by scanning with Shimadzu scanner and analyzing with Winrhizo software (Winrhizo, Regent Ltd, Canada).

### Plant vigor score by visual method

Plant vigor score was estimated by visual eye basis and it accounted for the number of branches, plant height, canopy size, and leaflet size, on a scale from 1 to 5 (1, low vigor, 5, high vigor). The score of parental genotype fell within that scale [high vigor ICC 4958 scored 5, whereas low vigor ICC 1882 scored 2), and progenies were falling between these two scores. This plant vigor score was done 20 DAS after sowing. All four replications were scored by one person eye visual score.

### Statistical analysis

The transpiration response to increasing VPD in the growth chamber was analyzed with non-linear regression of Graph pad Prism version 6 (Graph pad software, Inc., CA, and USA), which provides an r^2^ for the overall fit and slope values. For the response of transpiration to the inhibition of water transport pathways (Experiments 3 and 5), transpiration rate (TR) data were double normalized to a non-treated control. This consisted first in dividing the individual TR data by the mean TR of the control (TR ratio; TRR), and then by dividing the TRR values by the mean of TRR values before inhibitor treatment (Normalized TRR). The inhibition of water transport pathways (Aquaporin and apoplastic inhibition) and root hydraulic conductivity, canopy temperature estimation and transpiration rate under high VPD in contrasting TR and vigor groups along with parental lines were analyzed with statistical program package CoStat version 6.204 (Cohort Software, Monterey, CA, USA). One-way ANOVA was carried out to test for genotypic differences between the treatments and genotypes. Means were compared using Tukey-Kramer test and LSD (at *P* = 0.05). For this analysis, stable normalized TR values following the inhibitor treatment were used. For root hydraulic conductivity measurements, normalized root exudate values were used. Root exudate values were normalized against root surface area, pressure, and time (mg H_2_O cm^−2^min^−1^MPa^−1^) of both control and inhibited plants of each genotypes and replicates. For canopy temperature, mean values of both control (non-inhibited) and treatment (inhibited) were used. TR at high VPD and plant vigor score estimation experiment, mean TR, and vigor score data were used.

## Results

### TR response and plant vigor score in contrasting RILs and selection of the most contrasting materials

**Figure 1:**
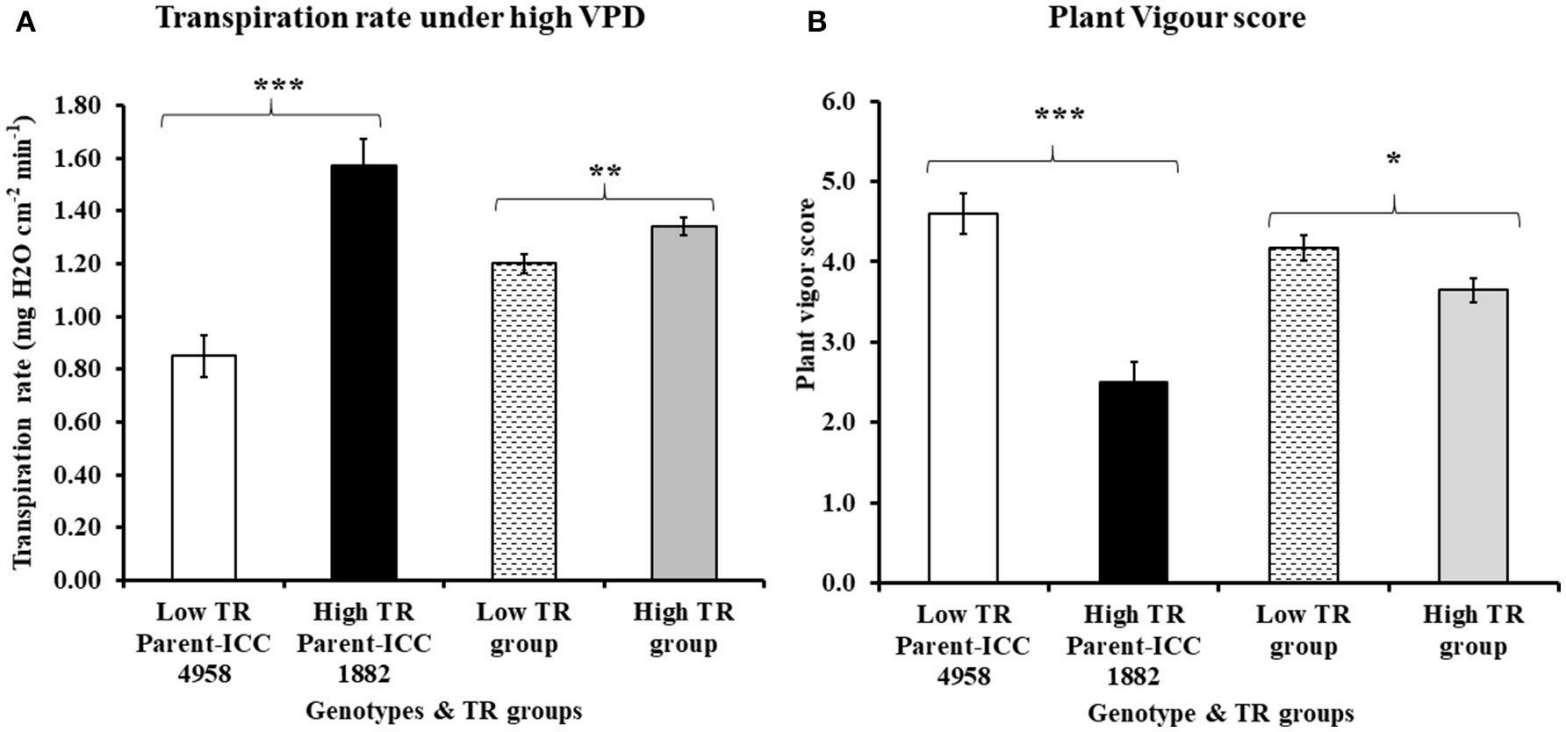
Variation in **(A)** transpiration rate under high VPD and **(B)** plant vigor score in both parents and progenies (RIL) contrasting TR group. In both graph A and B, open bar represents low TR parent ICC 4958, closed bar represents high TR parent ICC 1882, bar filled with broken lines represents the low TR group and bar filled with gray color represents the high TR group. Each data point of TR and plant vigor represents the means (±SE) of 6 and 4 replication respectively. Bars with ^*^, ^**^ and ^***^ (astric) symbols are significantly different from each other at *P* < 0.05, *P* < 0.01, and *P* < 0.001, respectively.

Variation in TR was observed among tested RIL progenies under high VPD (4.0 kPa) conditions, ranging from 0.945 ± 0.009 to 1.454 ± 0.041 (mg H_2_O cm^−2^min^−1^) (Table 1). The group of RILs that were selected initially with low TR under natural conditions had also lower TR than group of RILs that were selected with high TR under natural conditions, therefore matching observations made here under controlled conditions to earlier observations in natural conditions. The mean TR value of low TR group (1.20 ± 0.035 mg H_2_O cm^−2^min^−1^) was lower than in the high TR group (1.34 ± 0.033 mg H_2_O cm^−2^min^−1^) (Table 1). TR differences in contrasting groups and parental lines were statistically significant at *P* < 0.001 and *P* < 0.01, respectively (Figure 1A). Among the selected progenies, the highest TR (1.454 ± 0.041 mg H_2_O cm^−2^min^−1^) was for RIL 022 and the lowest TR was observed in RIL 211 (0.945 ± 0.009 mg H_2_O cm^−2^min^−1^) (Table 1), and these two RILs were selected for further assessments.

**Table 1:** Details of mean transpiration rate (TR; mg H_2_O cm^−2^ min^−1^) and plant vigor score in chickpea genotypes contrasting for plant vigor and VPD response.

**Sl.No**	**RIL No**.	**Groups**	**TR**	**SE**	**Vigor**	**SE**
1	**211**	**Low TR**	**0.945**	**0.009**	**4.25**	**0.40**
2	34	Low TR	1.136	0.009	4.00	0.29
3	221	Low TR	1.14	0.013	4.00	0.41
4	46	Low TR	1.153	0.016	4.00	0.25
5	199	Low TR	1.209	0.009	4.75	0.25
6	151	Low TR	1.232	0.011	5.00	0.40
7	207	Low TR	1.253	0.006	4.00	0.25
8	52	Low TR	1.265	0.009	3.25	0.25
9	128	Low TR	1.311	0.007	4.00	0.00
10	125	Low TR	1.319	0.012	4.50	0.29
11	20	High TR	1.139	0.022	3.50	0.29
12	161	High TR	1.188	0.014	4.00	0.25
13	35	High TR	1.301	0.051	3.75	0.25
14	217	High TR	1.326	0.038	4.00	0.48
15	144	High TR	1.338	0.017	4.50	0.29
16	123	High TR	1.37	0.041	2.75	0.25
17	90	High TR	1.38	0.045	3.50	0.50
18	224	High TR	1.433	0.016	3.75	0.25
19	170	High TR	1.439	0.051	3.25	0.48
20	**22**	**High TR**	**1.454**	**0.041**	**3.50**	**0.25**
21		**Mean-Low TR group**	**1.2**	**0.035**	**4.18**	**0.154**
22		**Mean-High TR group**	**1.34**	**0.033**	**3.65**	**0.15**

*It includes TR under high VPD (4.0 kPa) and plant vigor score estimated on a visual basis. Each data points of TR and plant vigor represent mean of six and four replication respectively. The letters SE denotes standard error and bold letters indicates selected lines (RIL 211 and RIL 022) contrasting for TR and vigor*.

Variation (2.75 ± 0.25 to 5.0 ± 0.40) in plant vigor score for contrasting TR groups was also observed (Table 1). Very clearly, low TR and high TR lines could be discriminated on the basis of their vigor. Indeed most of the high TR lines had low vigor, whereas most of the low TR lines had high vigor, except one (RIL52). The groups of RILs that were selected with low TR had higher vigor score (4.18 ± 0.154) than the groups of RILs selected with high TR (3.65 ± 0.150) (Table 1). Vigor differences in contrasting groups and parental lines were statistically significant (*P* < 0.001 and *P* < 0.05, respectively) (Figure 1B). Selected contrasting low TR line RIL 211 had higher vigor score (4.25 ± 0.40) than high TR line RIL 022 (3.50 ± 0.25) (Table 1).

### Transpiration rate (TR) response to increasing VPD in selected RILs and parents

**Figure 2:**
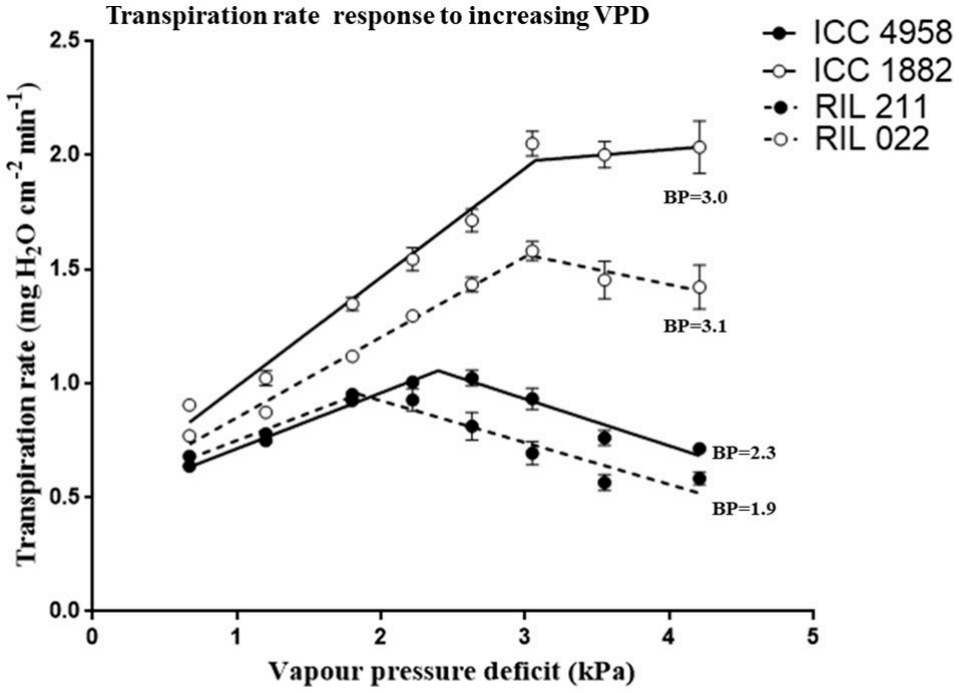
Transpiration rate (TR mg cm^−2^ min^−1^) response to increasing VPD conditions in four chickpea genotypes contrasting for plant vigor. The early vigor genotypes ICC 4958 (solid line with closed symbols) and RIL 211 (dashed line with closed symbols) showed earlier or lower VPD breakpoint values (2.3 and 1.9 kPa). The late vigor genotypes ICC 1882 (solid line with open symbols) and RIL 022 (dashedline with open symbols) showed higher VPD breakpoint (BP) values (3.0 and 3.1 kPa). Each data point represents the means (±SE) of 10 replicates per genotype.

There was a difference in TR between parental genotypes (ICC 4958 and ICC 1882) and between RILs (RIL 211 and RIL 022). The early vigor parental genotype ICC 4958 had lower TR than the late vigor parental genotype ICC 1882. Similarly, early vigor genotype RIL 211 had lower TR than late vigor RIL 022 (Figure 2). In both cases, the TR differences between the low and high vigor genotypes were larger under high VPD conditions. The mean TR over the entire range of VPD conditions were higher in late vigor parental genotype ICC 1882 (1.58 ± 0.16 mg H_2_O cm^−2^min^−1^) than in the early vigor parental genotype ICC 4958 (0.84 ± 0.05 mg H_2_O cm^−2^min^−1^). Similarly, early vigor progeny genotype RIL 022 mean TR (1.24 ± 0.10 mg H_2_O cm^−2^min^−1^) was higher than in late vigor progeny genotype RIL 211 (0.75 ± 0.05 mg H_2_O cm^−2^min^−1^) (Table 2). The parents and progenies were fitted with two straight line models (segmental regression analysis) with VPD breakpoints. The early vigor parental genotype ICC 4958 had lower VPD breakpoint (2.4 kPa) than late vigor parental genotype ICC 1882 (3.1 kPa). Similarly, late vigor progeny genotype of RIL 211 had earlier VPD breakpoints (1.8 kPa) than late vigor progeny genotype RIL 022 (3.08 kPa) (Figure 2 and Table 2). The early vigor progeny genotype RIL 211 had earlier VPD breakpoints than early vigor parental genotype ICC 4958, and also after the VPD breakpoints, TR was gradually decreased. This TR response pattern was closer to early vigor parent ICC 4958. The late vigor genotypes ICC 1882 and RIL 022 showed similar VPD breakpoints, and also TR was maintained after the breakpoints. This TR pattern response was closer to late vigor parent ICC 1882 (Figure 2).

**Table 2:** Regression (non-linear or segmental) results for the transpiration rate response to increasing VPD in the growth chamber.

**Genotypes**	**Mean TR and SE**	**Break point**	**Slope a**		**Slope b**		***R*^2^**
	**(mg H_2_O cm^2^ min^−1^)**	**Value (kPa)**	**Value (mg H_2_O cm^−2^ min^−1^)**	**SE**	**Value (mg H_2_O cm^−2^ min^−1^)**	**SE**	
ICC 4958	0.84 ± 0.05	2.39	0.2450	0.030	−0.2050	0.030	0.018
ICC 1882	1.58 ± 0.16	3.07	0.4780	0.036	0.0518	0.153	0.920
RIL 211	0.75 ± 0.05	1.84	0.2400	0.073	−0.1830	0.037	0.252
RIL 022	1.24 ± 0.10	3.01	0.3520	0.026	−0.1310	0.048	0.777
							

*Four chickpea [2 parents (ICC 4958 and ICC 1882) and 2 progenies (RIL 211 and RIL 22)] genotypes contrasting for plant vigor. The letters SE denotes standard error*.

### Effect of AQPs inhibitors on transpiration

**Figure 3:**
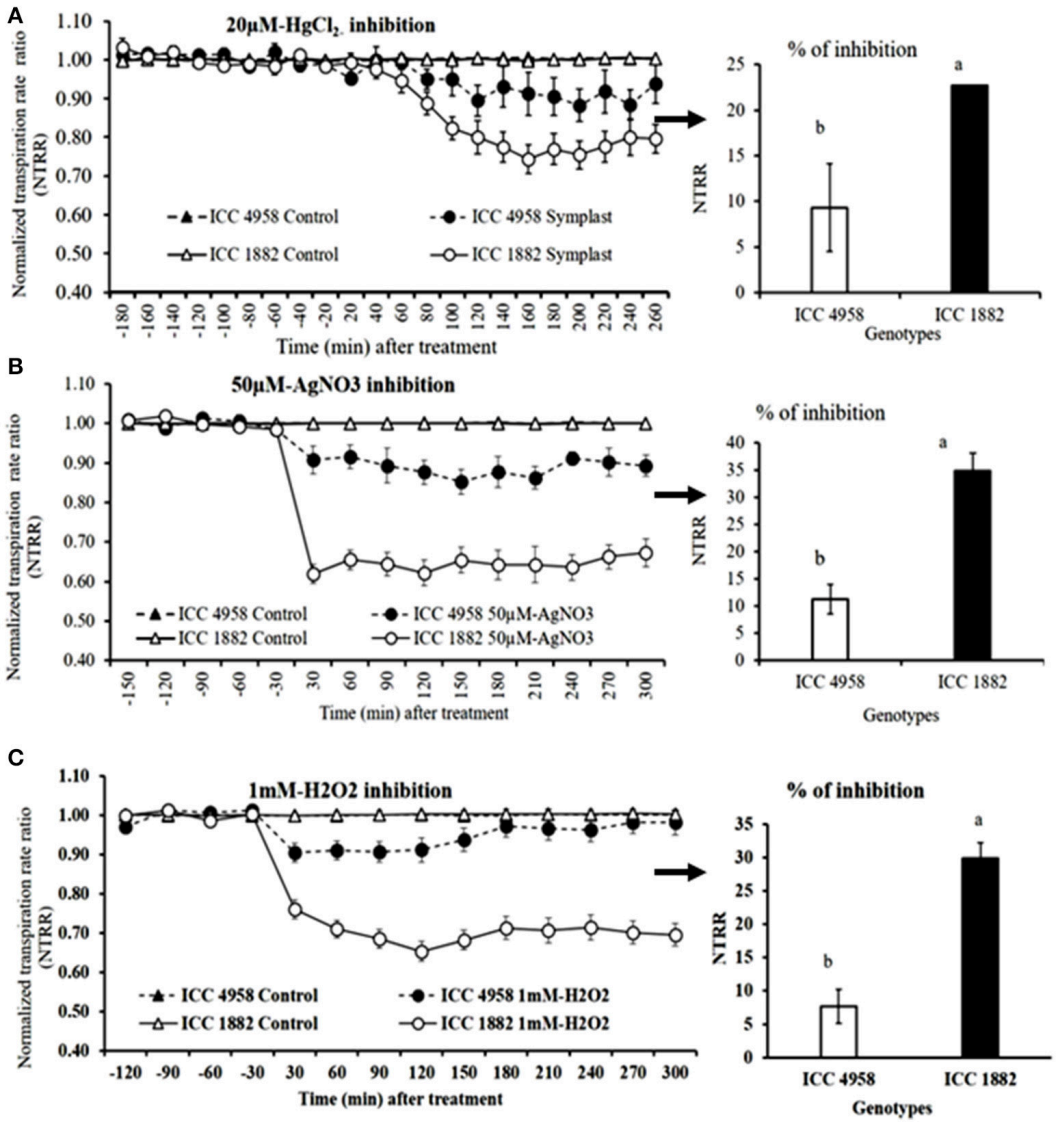
Decline in transpiration rate of two chickpea genotypes contrasting for plant vigor to the aquaporin inhibitors; 20 μM-HgCl_2_, 50 μM-AgNO_3_, and 1 mM-H_2_O_2_. The dashed lines with round shaped closed symbols represent ICC 4958 and the solid line with round shaped open symbols represent ICC 1882 genotype. The graph **(A)** represents the inhibition with 20 μM-HgCl_2_ and graph **(B)** represents the inhibition with 50 μM-AgNO_3_. The graph **(C)** represents the inhibition with 1 mM-H_2_O_2_. The arrows with respective bar graph represents % of inhibition, open bar represents, ICC 4958 and closed bar represents ICC 1882. Bars with different letters are significantly different (*P* < 0.05). Each treatment's data points represent the NTRR means (±SE) of eight replicates per genotype.

**Figure 4:**
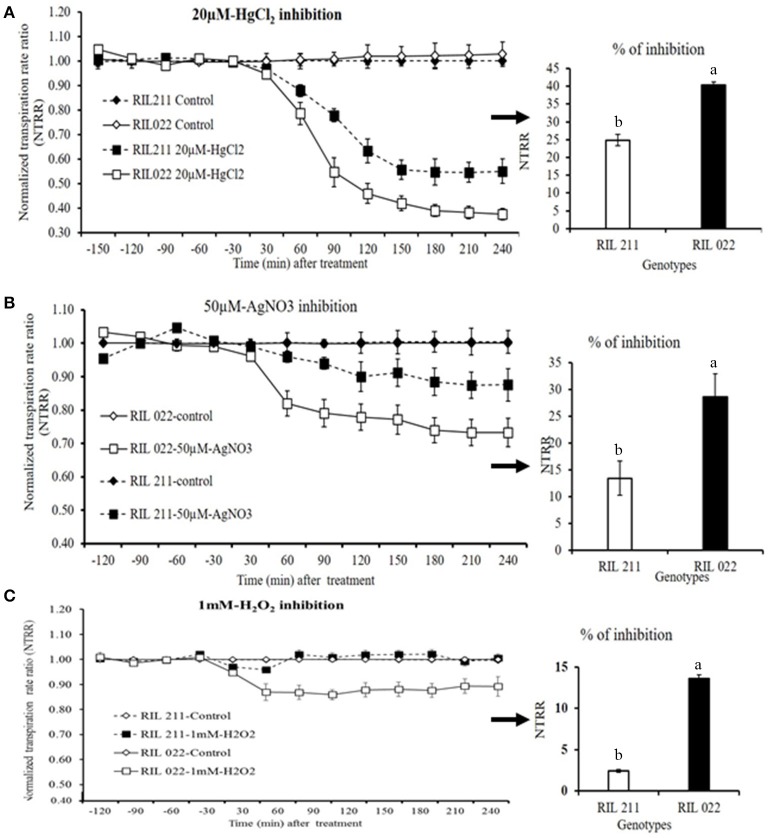
Decline in transpiration of two chickpea genotypes contrasting for plant vigor in response to the aquaporin inhibitors 20 μM-HgCl_2_, 50 μM-AgNO_3_, and 1 mM-H_2_O_2_. The solid line with open square symbols represents RIL 022 and the dashed lines with closed square symbols represents RIL 211. The graph **(A)** represents the inhibition with 20 μM-HgCl_2_ and graph **(B)** represents the inhibition with 50 μM-AgNO_3_. The graph **(C)** represents the inhibition with1 mM-H_2_O_2_. The arrows with respective bar graph represents % of inhibition (a, RIL 022; b, RIL 211), open bar represents ICC 4958 and closed bar represents ICC 1882. Bars with different letters are significantly different (*P* = 0.05). Each treatment's data points represent the NTRR means (±SE) of eight replicates per genotype.

When whole plants were treated with AQP inhibitors [H_2_O_2_ (1 mM), AgNO_3_ (50 μM) and HgCl_2_ (20 μM)], NTRR (Normalized transpiration rate ratio, NTRR) decreased in both parents and progenies. The late vigor/high TR parental genotype ICC 1882 had a higher transpiration inhibition than the early vigor/low TR parental genotype ICC 4958 with all three inhibitors (Figures 3A–C). In addition, the late vigor/high TR genotypes had a higher transpiration inhibition upon HgCl_2_ treatment under high (3.1 kPa) VPD condition. Similarly, the late vigor/high TR progeny genotype RIL 022 had higher NTRR inhibition than the early vigor/low TR progeny genotype RIL 211(Figures 4A–C. The maximum inhibition occurred after about 120 min of exposure to aquaporin inhibitors (H_2_O_2_ (1 mM), AgNO_3_ (50 μM), and HgCl_2_ (20 μM), with transpiration decreasing by 30, 35, and 22.7% in ICC 1882 and 7.6, 11.2, and 9.3% of ICC 4958. Similar trend was observed in progenies with a NTRR reduction of 13.6, 28.5, and 40.4% (H_2_O_2_ (1 mM), AgNO_3_ (50 μM), and HgCl_2_ (20 μM) in late vigor RIL 022, in contrast to only 3, 13.4, and 25% in early vigor/low TR RIL 211. The inhibition differences between genotypes were statistically significant for 1 mM-H_2_O_2_ at *P* < 0.1%, 50 μM-AgNO_3_ at *P* < 0.1%, 50 μM-AgNO_3_ at *P* < 0.1%. and 20 μM-HgCl_2_ at *P* < 0.05 level in parents and *P* < 0.1% (1 mM-H_2_O_2_), *P* < 0.05 (50 μM-AgNO_3_), and *P* < 0.1% (20 μM-HgCl_2_) level in progenies.

### Measurement of canopy temperature with AQP inhibition

**Figure 5:**
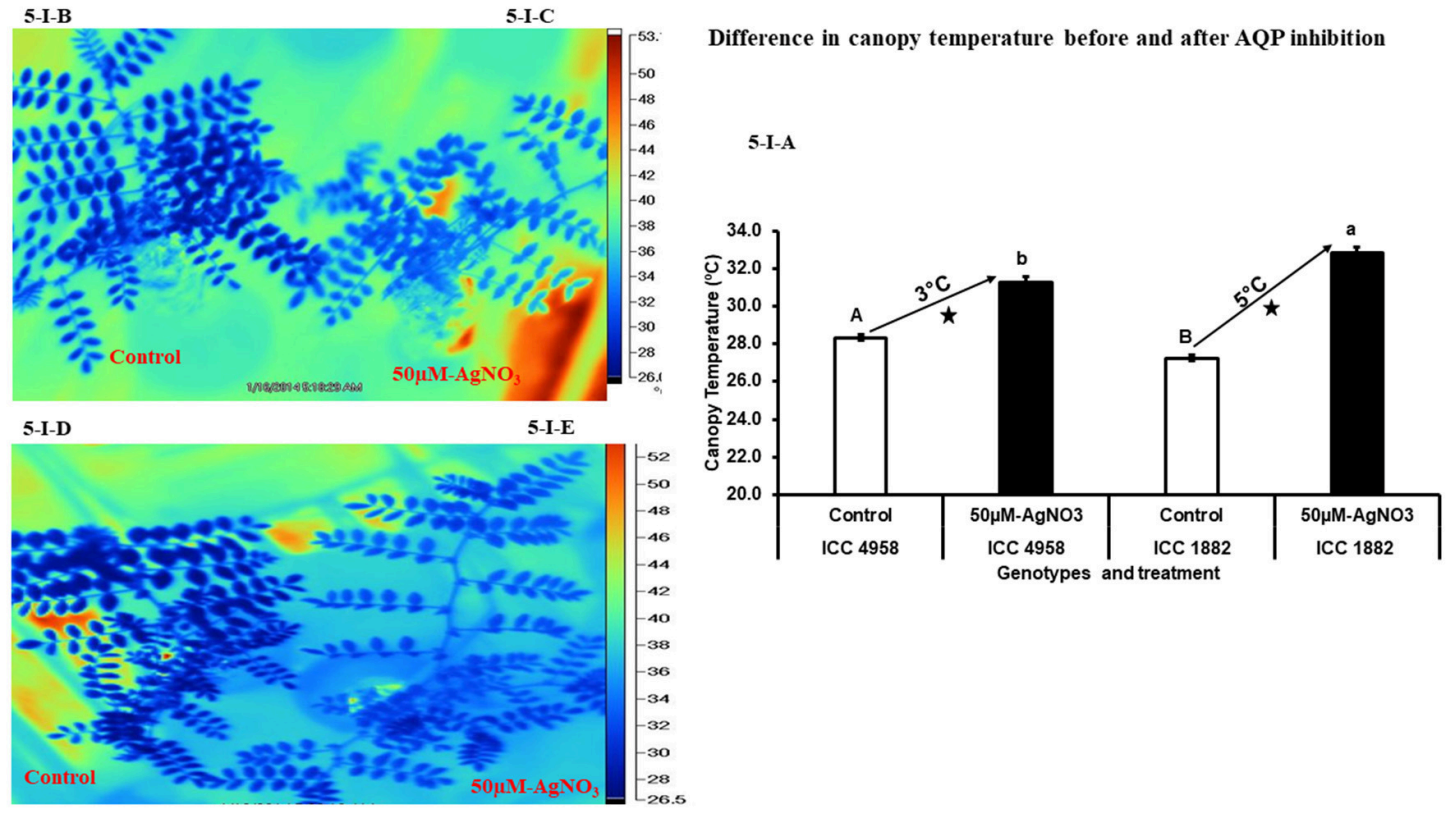
Variation in canopy temperature after treatment with aquaporin inhibitor (50 μM-AgNO_3_) for two chickpea parental genotypes contrasting for plant vigor. In the picture dark blue represents the control and light blue represents the treated plants. An open and closed bar indicates the canopy temperature of control and treated plants. The picture **(5-I-A)** represents silver inhibition and canopy temperature in non-inhibited (control) and inhibited (50 μM-AgNO_3_ treated) plants. Bars with different capital letters (A and B) and small letters (a and b) indicate significant differences (*P* < 0.05) in non-inhibited (control) and inhibited (treated) plants. The symbol star indicates the significantly different (*P* < 0.05) between non-inhibited (control) and inhibited (treated) plants. The picture (**5-I-B)** represents late vigor genotype ICC 1882 control, (**5-I-C)** represents late vigor genotype ICC 1882 treated, (**5-I-D)** represents early vigor genotype ICC 4958 control and **(5-I-E)** represents early vigor genotype ICC 4958 treated.

After treating the whole plants with AQPs inhibitor (50 μM-AgNO_3_) for 3 h, canopy temperature was measured in both control and treated plants. In control plants of both parental genotypes, the temperature was 27 ± 0.14°C in ICC 1882 and 28 ± 0.16°C in ICC 4958 (Figures 5-I-A,B,D). After plants were treated with 50 μM-AgNO_3_ inhibitor, canopy temperature of both genotypes increased. However, the late vigor genotype ICC 1882 had a higher (32 ± 0.30°C) increase in canopy temperature than the early vigor genotype ICC 4958 (31 ± 0.30°C) (Figures 5-I-A,C,E). The differences in increment of canopy temperature (after inhibition) between the genotypes were statistically significant at *P* = 5% level upon AgNO_3_ inhibition.

### Effect of apoplastic inhibitor on transpiration

**Figure 6:**
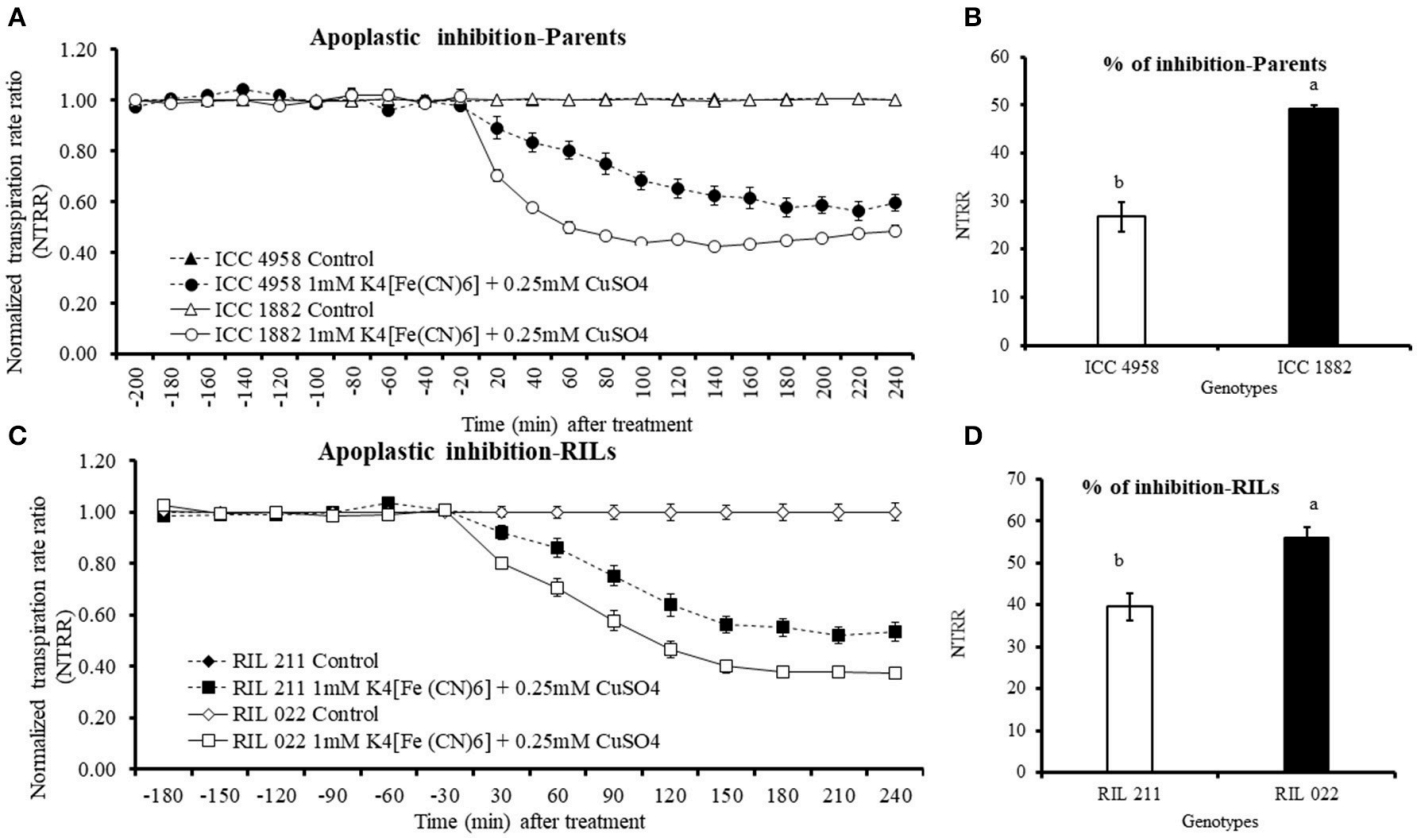
Decline in transpiration rate of four chickpea genotypes contrasting for plant vigor in response to the apoplastic inhibitor [1 mM K_4_[Fe(CN)_6_] and 0.25 mM- CuSO_4_]. The dashed lines with round shaped closed symbols represent ICC 4958 and solid line with round shaped open symbols represent ICC 1882 genotype. The solid lines with open square symbols represent RIL 022 and dashed lines with closed square symbols represent RIL 211 genotype. The graph **(A)** represents apoplastic inhibition of parents and graph **(B)** represents percentage of apoplastic pathway blocked. The graph **(C)** represents apoplastic inhibition of progenies (RILs) and graph **(D)** represents percentage of apoplastic pathway blocked. Each treatment data points represent the NTRR means (±SE) of eight replicates per genotype. Bars with different letters are significantly different (*P* = 0.05).

Transpiration was measured at 3.1 kPa (high VPD conditions) in both parents and progenies. Addition of 1 mM K_4_[Fe(CN)]_6_ and 0.25 mM CuSO_4_ resulted in rapid decrease of transpiration in all treated plants (Figures 6A,C). The late vigor/high TR genotype ICC 1882 and RIL 022 showed a higher and faster reduction of transpiration than early vigor/low TR genotype ICC 4958 and RIL 211 (Figures 6A,C). Overall, the level of NTRR reduction in parental and progenies genotypes to apoplastic inhibitor was 26.7 and 39.5% in early vigor low TR ICC 4958 and RIL 211, lower than the 50 and 56% in late vigor/high TR ICC 1882 and RIL 022 (Figures 6B,D). Both parents as well as progenies of genotypes showed clear genotypic difference with significant decreases after treatment with CuSO_4_. The inhibition difference between the genotypes were statistically significant at *P* = 0.1% level for parents and *P* = 1% level for progenies.

### Effect of water transport pathway inhibitor on root hydraulic conductivity

**Figure 7:**
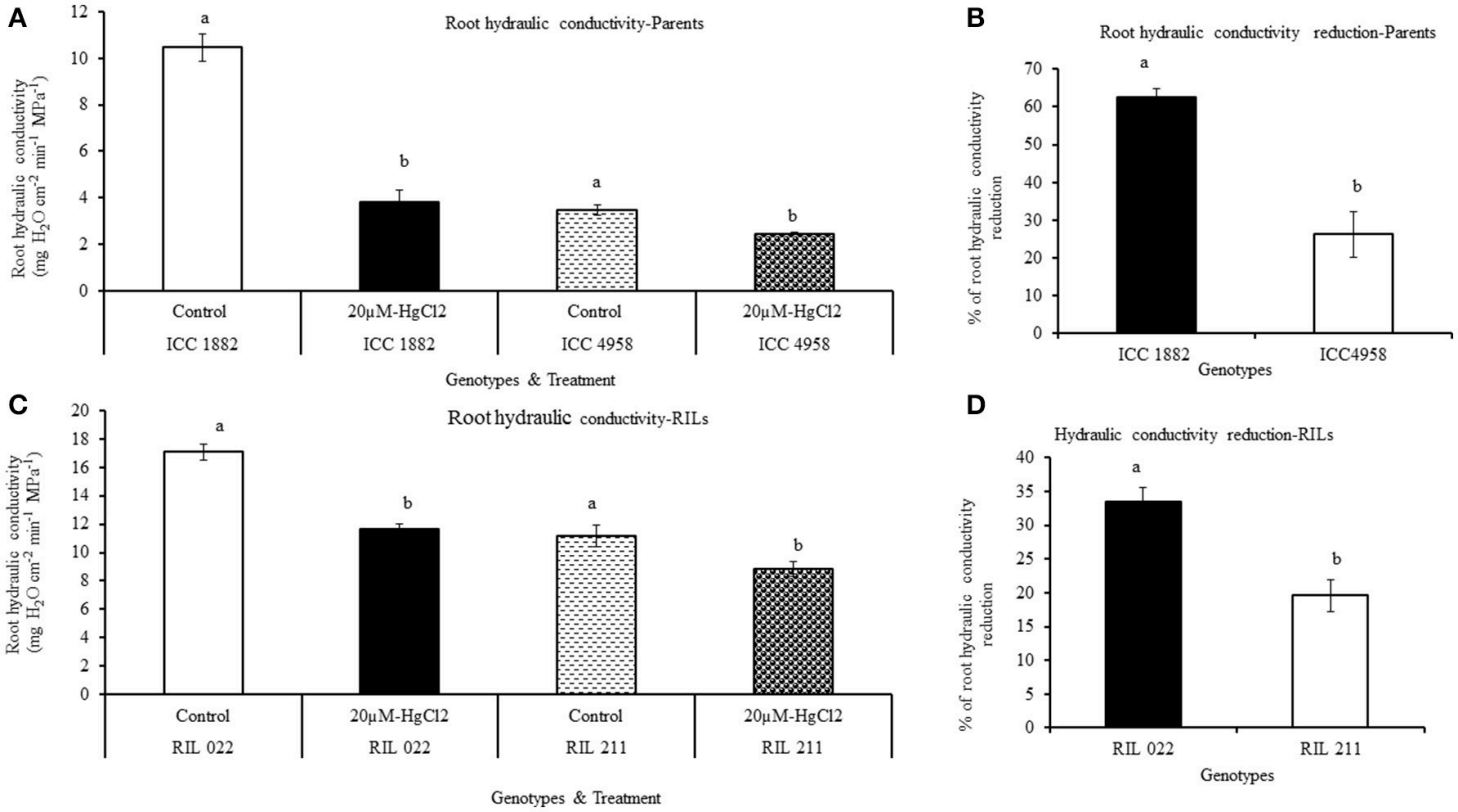
Differences root hydraulic conductivity in de-topped plants of four chickpea genotypes after aquaporin inhibition [20 μM-HgCl_2_]. Hydraulic conductivity under controlled and treated (inhibited) conditions were represents in graph **(A,C)** and also open bars represents ICC 1882 and RIL 022 and open bars filled with dashed lines represents ICC 4958 and RIL 211. The same graph **(A,C)** closed bars represent ICC 1882 and RIL 022 and closed bars filled with dotted spot represents ICC 4958 and RIL 211. The graph **(B,D)** represents percentage of hydraulic conductivity reduction parents and progenies. Each bar data represents the root hydraulic conductivity means (±SE) of eight replicates per genotype. Bars with different letters are significantly different (*P* = 0.05).

**Figure 8:**
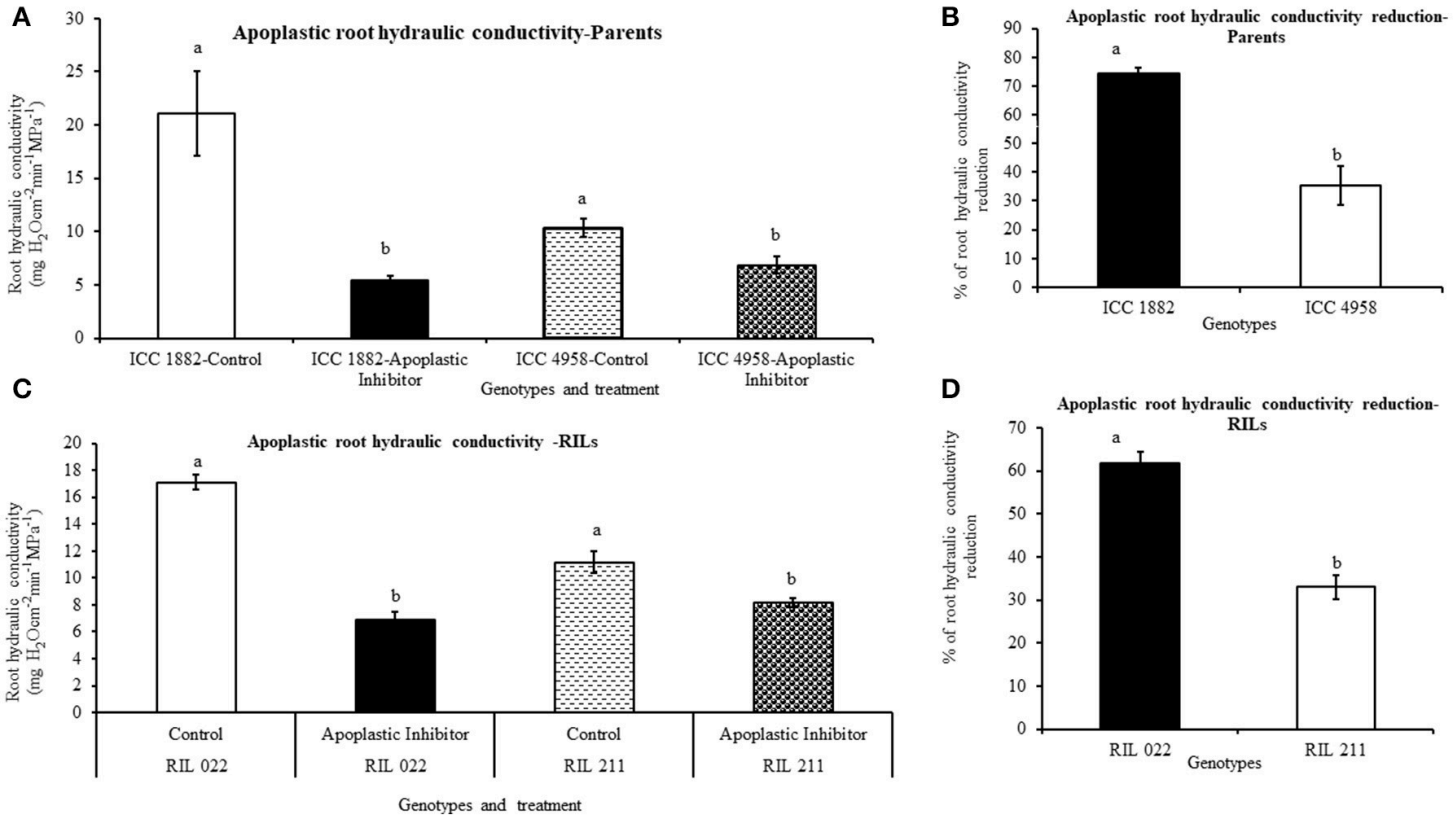
Differences root hydraulic conductivity in de-topped plants of four chickpea genotypes after apoplastic inhibition [1 mM K_4_[Fe(CN)_6_] and 0.25 mM CuSO_4_]. Hydraulic conductivity under controlled and treated (inhibited) conditions were represents in graph **(A,C)** and also open bars represents ICC 1882 and RIL 022 and open bars filled with dashed lines represents ICC 4958 and RIL 211. The same graph **(A,C)**, closed bars represents ICC 1882 and RIL 022 and closed bars filled with dotted spot represents ICC 4958 and RIL 211. The graph **(B,D)** represents percentage of hydraulic conductivity reduction parents and progenies. Each bar data represents the root hydraulic conductivity means (±SE) of eight replicates per genotype. Bars with different letters are significantly different (*P* = 0.05).

The root hydraulic conductivity was measured by pressure chamber method. In both parents and progenies, root hydraulic conductivity was measured after a 90 min treatment with the water transport pathway inhibitors (AQPs and apoplastic inhibitor), using non-treated plants as controls. Under non-treated conditions, early vigor/low TR parental genotype ICC 4958 had lower hydraulic conductivity than late vigor/high TR parental genotype ICC 1882 (Figures 7A,C, 8A,C). Similarly, early vigor/low TR progeny genotype RIL 211 had lower hydraulic conductivity than late vigor/high TR progeny genotype RIL 022 (Figures 7A,C).

When plant roots were treated with 20 μM-HgCl_2_ inhibitor, there was a rapid reduction of root hydraulic conductivity in all genotypes (Figures 7A–D). However, the % of root hydraulic conductivity reduction was higher (63 and 35.5%) in the late vigor/high TR parental and progeny genotype (ICC 1882 and RIL 022) than in the early vigor/low TR parental and progeny genotype (ICC 4958 and RIL 211) (32 and 19.6%, respectively) (Figures 7A–D). Similarly, when plant roots were treated with the apoplastic inhibitor (1 mM K_4_[Fe(CN)]_6_ and 0.25 mM CuSO_4_), there was also a rapid reduction of root hydraulic conductivity in all genotypes (Figures 8A–D). However, the % of root hydraulic conductivity reduction was higher (74 and 62%) in the late vigor/high TR parental and progeny genotype (ICC 1882 and RIL 022) than in the early vigor/low TR parental and progeny genotype (ICC 4958 and RIL 211) 35 and 33%, respectively (Figures 8A–D).

The differences in root hydraulic conductivity between genotype under treated and non-treated conditions were statistically significant at *P* < 0.1% (AQPs inhibition) and *P* = 1% (apoplastic inhibition) levels in parents and *P* = 1% (AQPs inhibition) and *P* < 0.1% (apoplastic inhibition) level progenies. It should also be mentioned that the decrease in root hydraulic conductivity was higher when the apoplast was inhibited than when the symplast was inhibited (Figures 7, 8).

### De-rooted shoot inhibition by aquaporin inhibitor (50 μM-HgCl_2_)

**Figure 9:**
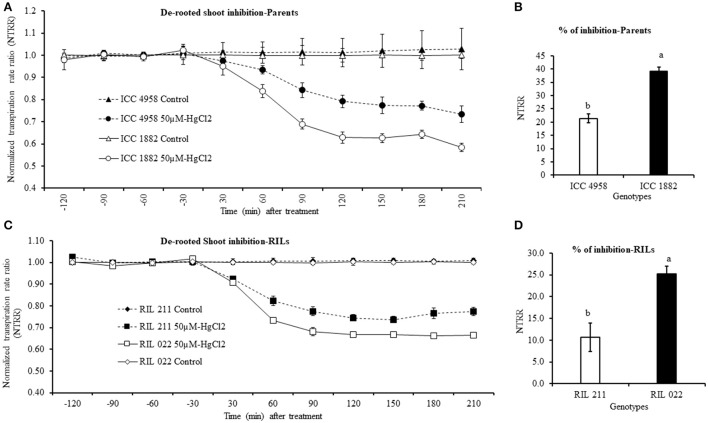
Decline in de-rooted shoot transpiration of four chickpea genotypes contrasting for plant vigor to the aquaporin inhibitor (50 μM-HgCl2). The graph **(A)** represents de-rooted shoot inhibition of parents. In this graph the dashed line with closed circle symbols represents ICC 4958 and the solid line with open circle symbols represents ICC 1882 genotype. The graph **(C)** represents de-rooted shoot inhibition of progeny (RILs). In this graph the dashed line with closed square symbols represents RIL 211 and the solid lines with open square symbols represent RIL 022 genotype. The graph **(B,D)** represents percentage of aquaporin inhibition in parents and progenies. Each treatment data points represent the NTRR means (±SE) of six replicates per genotype. Bars with different letters are significantly different (*P* = 0.05).

De-rooted shoot transpiration was measured at 2.5 kPa (moderate VPD) in both parents and progenies. When de-rooted shoot was treated with 50 μM-HgCl_2_, transpiration rapidly decreased in all tested genotypes. The late vigor/high TR parental and progeny genotype ICC 1882 and RIL 022 had a higher transpiration decline than early vigor/low TR ICC 4958 and RIL 211 (Figures 9A,C). The maximum inhibition occurred after about 120 min of exposure to 50 μM-HgCl_2_ with a transpiration decline of 39 and 22.5% in late vigor/high TR parental and progeny genotype (ICC 1882 and RIL 022) more than the 21.4 and 10.7% decrease in transpiration in the early vigor/low TR parental and progeny genotype (ICC 4958 and RIL 211) (Figures 9B,D). The differences in de-rooted shoot inhibition between genotype were statistically significant at 0.1% levels in parents and 1% level in progenies.

## Discussion

In summary of this work, there was a clear relationship between high TR and low vigor score, with a few exceptions to that trend. Then, early vigor/low TR lines had less transpiration inhibition than late vigor/high TR lines upon treatment with aquaporin inhibitors or blockers of the apoplast, and transpiration inhibition of de-rooted shoots followed a similar pattern. Early vigor/low TR lines had lower root hydraulic conductivity than late vigor/high TR lines under non-inhibited conditions. When the roots were treated with aquaporin inhibitors or blockers of the apoplast, early vigor/low TR lines had less reduction in root hydraulic conductivity than late vigor/high TR lines. Inhibiting the apoplastic pathway led to a larger reduction in transpiration and root hydraulic conductivity than the inhibition of the symplast pathway, suggesting a predominant importance of the apoplast in channeling water through the root cylinder. Overall, water transport pathway studies in whole plants, de-rooted shoot, and de-topped root revealed that vigor and TR under high VPD were closely related to one another and contrasts in these traits appeared to be driven by differences in their use of water transport pathways in the root cylinder, therefore linking plant hydraulic properties with traits involved in the adaptation to water stress.

### Early vigor genotypes restrict transpiration under high VPD conditions

Crop adaptation to water stress is a matter of matching water supply to crop water demand, especially by ensuring there is sufficient water available for the grain filling period (Vadez et al., 2013). Crop water management strategies, therefore, relate to traits that alter the balance between supply and demand and especially this balance during critical crop stages. Early reports (Cooper et al., 1987; Richards, 1996) suggested that plant vigor affects the radiation use efficiency and also helps in reducing soil evaporation, which then helps in maximizing the availability of soil water for transpiration. In addition, a crop modeling study revealed that greater early vigor had the potential to increase average grain yields by 8–10% in Mediterranean environments and in typical of parts of the Australian wheat belt (Condon et al., 2004). On the contrary, early vigor may also lead to an early depletion of soil moisture and a poorer seed filling, as shown earlier in chickpea and pearl millet (Zaman-allah et al., 2011b; Vadez et al., 2013). Therefore, transpiration restriction under increasing VPD, at different VPD thresholds, is also a mean for adapting to water stress conditions. Transpiration restriction under high VPD, as shown in Table 1 and Figure 1A, is indeed an important water conservation strategy (Sinclair et al., 2008) for which genotypic variation has been reported in other species such as pearl millet (*Pennisetum glaucum* L.; Kholova et al., 2010), sorghum (*Sorghum bicolor* L.; Gholipoor et al., 2010), soybean (*Glycine max* L.; Merr; Fletcher et al., 2007; Gilbert et al., 2011), peanut (*Arachis hypogaea* L.; Devi et al., 2010), cowpea (*Vigna unguiculata* L.; Belko et al., 2012), and recently in maize (*Zea mays* L.; Yang et al., 2012; Gholipoor et al., 2013).

In the present study, early plant vigor genotypes (ICC 4958 and RIL 211) usually had transpiration restriction under increasing VPD, and this restriction also took place at lower VPD thresholds. Therefore, the results presented here suggest that there is a breeding opportunity to combine these two positive traits into a single plant ideotype. Since there were exceptions to that relationship, it is also conceivable to design ideotypes that would have a different “dosage” of either traits.

### The vigor/TR relationship seen from the standpoint of aquaporin inhibition results

The relationship between vigor and the transpiration restriction under increasing VPD could be expected, i.e., larger canopy exposed to higher evaporative demand may have more difficulty in channeling sufficient water to support transpiration. On the contrary, it is known that root and shoot develop in a closely coordinated manner (Bouteillé et al., 2012), so that plants with a large canopy should be equally able to channel sufficient water to support transpiration under high evaporative demand than plants with small canopy. The fact that there was a vigor-transpiration restriction relationship suggests that something else than root/shoot development affects water supply to support transpiration under high evaporative demand. In other words, assuming large canopies are paralleled with large root systems, the genotypes having a transpiration restriction under high VPD might either not have a root system large enough to channel sufficient water to support transpiration or may have had a limitation in the capacity of roots to channel water. Indeed, chickpea genotypes contrasting for plant vigor/TR clearly discriminated in their utilization of water transport pathways. Upon aquaporin inhibition or apoplast blockage, early vigor/low TR genotypes (ICC 4958 and RIL 211) had only limited transpiration inhibition compared to late vigor/high TR genotype (ICC 1882 and RIL 022). A similar response pattern was observed in de-rooted shoots (shoot), when AQPs inhibitors were used. This would indicate that early vigor/low TR genotypes had a limited dependence on the aquaporin-mediated water transport pathway, which would decrease their capacity to channel water in the root cylinder, thereby this also potentially being a reason for their lower hydraulic conductivity. This whole plant inhibition results were in agreement with our earlier work in chickpea (Sivasakthi et al., 2017) and pearl millet (Tharanya et al., 2017), which showed low TR lines had lower TR than high TR lines after the inhibition of AQP mediated pathway. Similar findings were reported in wheat (Schoppach et al., 2013) and sorghum (Choudhary et al., 2013). The results from de-rooted shoot were in line with soybean (Sadok and Sinclair, 2010), peanut (Devi et al., 2012), and sorghum Choudhary et al., 2013, showing that when de-rooted shoots were fed with aquaporin inhibitor, low TR lines had less inhibition than high TR lines. Similar results were also reported in other crop species like barley (83–92%; Tazawa et al., 1997), wheat (75%; Maggio and Joly, 1995), and tomato (50%; Zhang and Tyerman, 1999) in which a large decrease in root hydraulic conductivity (Lpr) was observed in response to HgCl_2_ treatment.

In summary, lower TR under high VPD and transpiration restriction at early VPD breakpoints in early vigor/low TR lines is interpreted to be a consequence of a limited involvement of certain populations of aquaporins in water transport pathways, which could not be tuned up under conditions of high evaporative demand, which then led to transpiration breakpoint at lower VPD (below 2.4 kPa). In case of low vigor genotypes/high TR lines, their high VPD (above 3.0 kPa) breakpoints could be interpreted as an ability to meet the high transpiration demand by the increase in water uptake through the AQP-mediated pathway, resulting in a much larger transpiration rate than early vigor lines.

### What root hydraulics model for water transport in these contrasting lines?

Under controlled (non-inhibited) conditions, the early vigor/low TR genotypes ICC 4958 and RIL 211 had lower root hydraulic conductivity than the late vigor/high TR genotypes ICC 1882 and RIL 022. When treated with aquaporin inhibitors or blockers of the apoplast, late vigor/high TR parental and progeny genotype had higher reduction in root hydraulic conductivity than early vigor/low TR genotypes. Higher reduction in root hydraulic conductivity was observed with apoplastic inhibitors than AQP inhibitors. Our interpretation is that late vigor/high TR genotypes depended more on the apoplast for water transport than early vigor/low TR genotypes. The fact that their root hydraulic conductivity decreased less upon apoplastic blocker, but that their root hydraulic conductivity under non-inhibited condition was lower than the high TR lines, is intruiguing. The interpretation from the experiments on the apoplast suggest that the low TR genotypes may have a smaller apoplastic space for water transport than the high TR lines, which would be compensated by their known larger root system (Varshney et al., 2014), also explaining their lower root hydraulic conductivity. Such apoplastic features, together with the high root density of early vigor genotypes, would allow them large TR under moderate VPD conditions but would then, together with a lower dependence on the aquaporin-mediated pathways for water transport, allow TR restriction under high VPD. Of course, root anatomical work would be needed to test that hypothesis. These results were in agreement with our previous work in chickpea (Sivasakthi et al., 2017) and pearl millet (Tharanya et al., 2017). Similar results were reported in rice where it was shown that the relative contribution of the apoplastic path was much larger than that of the cell-to-cell path (aquaporin mediated) for overall radial water flow (Ranathunge et al., 2004).

In summary, these experimental results suggest a predominance of the apoplast in channeling water through the root cylinder, which could be tuned up under high VPD with the aquaporin-mediated water transport pathway in the case of late vigor/high TR lines. The low root hydraulic conductivity of early vigor/low TR genotypes would be compensated by their larger root system under low VPD conditions.

## Conclusion

This work showed a close linkage between vigor/TR and water transport pathways, which seemed to be related to differences in the degree of utilization of water transport pathways (AQPs mediated and apoplastic). The early vigor/low TR genotypes utilized the AQP-mediated water transport pathway less than the late vigor/high TR genotypes under high VPD conditions. The apoplastic water transport pathway predominated over the symplastic pathway. Overall, this work demonstrated a close linkage between features of the plant hydraulics characteristics and traits closely involved in the crop adaptation to water stress, opening an opportunity to link the genetics of key adaptive traits and their use in the breeding of improved varieties for water limited conditions.

## Author contributions

KS, MT and RW: Conducted the experiments. KS and MT: Analyzed and summarized the data. KS, JK and VV: Wrote the manuscript. VV, JK and TT: Manuscript review. VV: Conceived and directed the project. All authors read the MS and provided their consent. All authors read and approved the final manuscript.

### Conflict of interest statement

The authors declare that the research was conducted in the absence of any commercial or financial relationships that could be construed as a potential conflict of interest.
