# Supplementary material for: Chickpea Genotypes Contrasting for Vigor and Canopy Conductance Also Differ in Their Dependence on Different Water Transport Pathways
Source: Front Plant Sci. 2017 Sep 26;8:1663. doi: 10.3389/fpls.2017.01663 (PMC5649140; doi:10.3389/fpls.2017.01663)
Supplement: Supplementary file 1 [file Table1.docx]

**Supplementary table 1** Summary of experiments include genotypes, name of the experiment, plant growth system, measured part and trait, concentration and chemicals (inhibitors), replications and measured VPD.

| **Exp. No.** | **Genotypes** | **Name of the**  **experiment** | **Plant**  **growth system** | **Measured Part**  **and trait** | **Concentration and chemicals (inhibitors) used** | **No of replications per genotype** | **Measured VPD (kPa)** |
| --- | --- | --- | --- | --- | --- | --- | --- |
| 1 | 10 low TR RILs  10 high TR RILs | VPD response to TR | Black Soil (Vertisol) | Whole plant and TR (mg H_2_O cm^-2^min^-1^) | - | 6 | (1.3-4.0) |
| 2 | ICC 4958 & ICC1882  RIL 211 & RIL 022 | VPD response to TR | Black Soil (Vertisol) | Whole plant and TR (mg H_2_O cm^-2^min^-1^) | - | 12 | (0.7-4.21) |
| 3 | ICC 4958  ICC 1882 | Aquaporin (AQPs) inhibition | Hydroponics | Whole plant and % of aquaporin (AQPs) inhibition | 20µM-HgCl_2_ | 6 control  9 treatment | 3.1 |
|  | ICC 4958  ICC 1882 | Aquaporin (AQPs) inhibition | Hydroponics | Whole plant and % of aquaporin (AQPs) inhibition | 50µM-AgNO_3_ | 6 control  8 treatment | 3.1 |
|  | ICC 4958  ICC 1882 | Aquaporin (AQPs) inhibition | Hydroponics | Whole plant and % of aquaporin (AQPs) inhibition | 1mM-H_2_O_2_ | 6 control  9 treatment | 3.1 |
|  | RIL 211  RIL 022 | Aquaporin (AQPs) inhibition | Hydroponics | Whole plant and % of aquaporin (AQPs) inhibition | 1mM-H_2_O_2_ | 6 control  9 treatment | 3.1 |
|  | RIL 211  RIL 022 | Aquaporin (AQPs) inhibition | Hydroponics | Whole plant and % of aquaporin (AQPs) inhibition | 50µM-AgNO_3_ | 6 control  8 treatment | 3.1 |
|  | RIL 211  RIL 022 | Aquaporin (AQPs) inhibition | Hydroponics | Whole plant and level of aquaporin (AQPs) inhibition | 20µM-HgCl_2_ | 6 control  7 treatment | 3.1 |
|  | ICC 4958  ICC 1882 | Apoplastic inhibition | Hydroponics | Whole plant and % of apoplastic inhibition | 1mM-K4[Fe(CN)_6_] & 0.25mM-CuSO_4_ | 8 control  8 treatment | 3.1 |
|  | RIL 211  RIL 022 | Apoplastic inhibition | Hydroponics | Whole plant and  % of apoplastic inhibition | 1mM-K4[Fe(CN)_6_] & 0.25mM-CuSO_4_ | 6 control  6 treatment | 3.1 |
| 4 | ICC 4958  ICC 1882 | Root hydraulic conductivity with AQP inhibition | Hydroponics | Detached Root and Root hydraulic conductivity &  (mg H_2_O cm^-2^ min^-1^ MPa^-1^)  % of Root hydraulic conductivity reduction | 20µM-HgCl_2_ | 8 control  8 treatment | 1.5-2.5 |
|  | ICC 4958  ICC 1882 | Root hydraulic conductivity with apoplastic inhibition | Hydroponics | Detached Root and Root hydraulic conductivity &  (mg H_2_O cm^-2^ min^-1^ MPa^-1^)  % of Root hydraulic conductivity reduction | 1mM-K4[Fe(CN)_6_] & 0.25mM-CuSO_4_ | 8 control  10 treatment | 1.5-2.5 |
|  | RIL 211  RIL 022 | Root hydraulic conductivity with AQP inhibition | Hydroponics | Detached Root and Root hydraulic conductivity &  (mg H_2_O cm^-2^ min^-1^ MPa^-1^)  % of Root hydraulic conductivity reduction | 20µM-HgCl_2_ | 8 control  8 treatment | 1.5-2.5 |
|  | RIL 211  RIL 022 | Root hydraulic conductivity with Apoplastic inhibition | Hydroponics | Detached Root and Root hydraulic conductivity &  (mg H_2_O cm^-2^ min^-1^ MPa^-1^)  % of Root hydraulic conductivity reduction | 1mM-K4[Fe(CN)_6_] & 0.25mM-CuSO_4_ | 8 control  10 treatment | 1.5-2.5 |
| 5 | ICC 4958  ICC 1882 | De-rooted Shoot inhibition | Black Soil (Vertisol) | De-rooted shoot and % of aquaporin (AQPs) inhibition | 50µM-HgCl_2_ | 5 control  6 treatment | 2.5 |
|  | RIL 211  RIL 022 | De-rooted Shoot inhibition | Black Soil (Vertisol) | De-rooted shoot and % of aquaporin (AQPs) inhibition | 50µM-HgCl_2_ | 6 control  6 treatment | 2.5 |

**Supplementary table 2.** Details of vapour pressure deficit (VPD) stress imposition inside the growth chamber.

| **Sl.No** | **Temperature (°C)** | **Relative Humidity (RH %)** | **Photosynthetic photon flux density**  **(PPFD; µmol m^-2^ s^-1^)** | **Vapour pressure deficit (VPD; kPa)** |
| --- | --- | --- | --- | --- |
| 1 | 27 | 75 | 450 | 0.9 |
| 2 | 29 | 65 | 450 | 1.4 |
| 3 | 31 | 55 | 450 | 2.0 |
| 4 | 33 | 47 | 450 | 2.7 |
| 5 | 35 | 45 | 450 | 3.0 |
| 6 | 36 | 40 | 450 | 3.6 |
| 7 | 37 | 33 | 450 | 4.2 |
